# Absence of Tissue Inhibitor of Metalloproteinase-4 (TIMP4) ameliorates high fat diet-induced obesity in mice due to defective lipid absorption

**DOI:** 10.1038/s41598-017-05951-4

**Published:** 2017-07-24

**Authors:** Siva S. V. P. Sakamuri, Russell Watts, Abhijit Takawale, Xiuhua Wang, Samuel Hernandez-Anzaldo, Wesam Bahitham, Carlos Fernandez-Patron, Richard Lehner, Zamaneh Kassiri

**Affiliations:** 1 Department of Physiology, Faculty of Medicine and Dentistry, University of Alberta, Edmonton, AB Canada; 2 Department of Biochemistry, Faculty of Medicine and Dentistry, University of Alberta, Edmonton, Alberta Canada; 3 Group on Molecular and Cell Biology of Lipids, Faculty of Medicine and Dentistry, University of Alberta, Edmonton, Alberta Canada

## Abstract

Tissue inhibitor of metalloproteases (TIMPs) are inhibitors of matrix metalloproteinases (MMPs) that regulate tissue extracellular matrix (ECM) turnover. TIMP4 is highly expressed in adipose tissue, its levels are further elevated following high-fat diet, but its role in obesity is unknown. Eight-week old wild-type (WT) and *Timp4*-knockout (*Timp4* ^−/−^) mice received chow or high fat diet (HFD) for twelve weeks. *Timp4* ^−/−^ mice exhibited a higher food intake but lower body fat gain. Adipose tissue of *Timp4* ^*−/–*^-HFD mice showed reduced hypertrophy and fibrosis compared to WT-HFD mice. *Timp4* ^*−/–*^-HFD mice were also protected from HFD-induced liver and skeletal muscle triglyceride accumulation and dyslipidemia. *Timp4* ^−/−^-HFD mice exhibited reduced basic metabolic rate and energy expenditure, but increased respiratory exchange ratio. Increased free fatty acid excretion was detected in *Timp4* ^−/−^-HFD compared to WT-HFD mice. CD36 protein, the major fatty acid transporter in the small intestine, increased with HFD in WT but not in *Timp4* ^−/−^ mice, despite a similar rise in *Cd36* mRNA in both genotypes. Consistently, HFD increased enterocyte lipid content only in WT but not in *Timp4* ^−/−^ mice. Our study reveals that absence of TIMP4 can impair lipid absorption and the high fat diet-induced obesity in mice possibly by regulating the proteolytic processing of CD36 protein in the intestinal enterocytes.

## Introduction

Obesity is a pathophysiological state involving excess accumulation of triglycerides in the adipose tissue, thereby increasing the risk of developing fatty liver, dyslipidemia, insulin resistance and hypertension^1–4^. In particular, visceral obesity associated with these complications constitute metabolic syndrome which in turn increases the risk of type II diabetes and cardiovascular diseases^5^. Identifying the molecular mechanism involved in development of obesity and the associated complications will provide insight into developing novel therapeutic strategies for this syndrome.

During energy-rich conditions, adipose tissue stores the energy in the from of triglycerdies (TG), and upon systemic energy demand, adipose tissue TG undergoes lipolysis and free fatty acids (FFAs) are released into the circulation and then used as an energy source. Although initially adipose tissue was believed to be an energy storing organ, recently it has been shown to secrete various signaling molecules named “adipokines” through which adipose tissue communicates with other organs to maintain systemic energy metabolism^6^. Excess storage of TG in adipose tissue during obesity alters its metabolic and secretive functions leading to development of obesity-associated comorbidities^7^. As in other organs, extracellular matrix (ECM) plays a critical role in maintaining the structure and function of the adipose tissue^8^. Adipose tissue is a very dynamic organ with various cell types, including adipocytes, pre-adipocytes, immune cells and the vasculature. Preadipocyte differentiation, adipocyte growth, vasculature formation and immune cell flux involves dynamic remodeling of the ECM^8^, which provides structural support for the tissues as well as regulating cell functions by activating various signaling mechanisms directly or indirectly by sequestering signaling molecules^9^.

Tissue inhibitor of metalloproteinases (TIMPs) are best known as endogenous inhibitors of matrix metalloproteinases (MMPs) and disruption of the MMP-TIMP balance influences the rate of ECM turnover^10^. A number of MMPs (e.g. MMP2, MMP9, and MMP13) and the four TIMPs (TIMP1–4) are expressed in the adipose tissue^11, 12^, and they have been reported to be involved in development of obesity and associated complications^13–16^. TIMP1 has been reported to be an adipokine which is upregulated in adipose tissue under obese conditions, increases preadipocyte differentiation and adipocyte fat accumulation and can be used as a predictor of adiposity in humans^17–19^. *Timp1*-deficient mice are resistant to diet-induced obesity^20^, whereas *Timp2*-knockout mice are prone to diet-induced obesity^21^. Concomitant deficiency in *Timp3* and insulin receptor resulted in adipose tissue inflammation and fatty liver, whereas macrophage-specific TIMP3 overexpression was protective against insulin resistance^22, 23^.

TIMP4 is highly expressed in the adipose tissue^24^, however the role of TIMP4 in adipose tissue biology and obesity has not been explored so far. In this study, we investigated the role of TIMP4 in obesity using *Timp4*-deficient mice and found that TIMP4 promotes high fat-induced obesity, fatty liver and dyslipidemia possibly by promoting intestinal lipid absorption by preventing proteolytic processing of CD36, the cell surface fatty acid transporters in enterocytes.

## Materials and Methods

### Animal experiment

Heterozygous *Timp4* knock-out mice (C57BL/6 background; Texas A&M Institute for Genomic Medicine) were bred to generate wild-type (WT) and *Timp4* knock-out mice (*Timp4* ^−/−^). Eight weeks old male mice from each genotype were fed either chow-diet (10% calories from fat; D10012M from Research diets) or high-fat diet (HFD, 60% calories from fat; TD06414 from Envigo) *ad libitum* for twelve weeks. Body weights were measured weekly and cumulative food intake was measured weekly twice. Body composition analysis, basic metabolic rate studies and glucose tolerance tests were performed between 10^th^ and 12^th^ week of the study. At 12 weeks, mice were euthanized and tissues were collected after overnight fasting (twelve hours) or in fed-sate. Blood was collected by cardiac puncture, plasma and various tissues were stored at −80 °C for further analyses, or formalin-fixed and paraffin-embedded for histological analysis. All animal procedures were approved by the University of Alberta Animal Care and Use Committee, and in accordance with guidelines of the Canadian Council on Animal Care.

### Body composition analysis

Total body fat mass and lean mass were determined by using Echo-MRI (Echo Medical Systems, Houston, Texas). Fat mass and lean mass percentages were calculated from the whole-body weights.

### Basic Metabolic Rate (BMR)

Basic metabolic rate (VO_2_ consumption), VCO_2_ release and energy expenditure (EE) were measured by indirect calorimetry for 24 hours in wild-type and TIMP4KO mice belonging to both chow-fed and high-fat-fed groups (Comprehensive Laboratory Animal Monitoring System, Columbus Instruments, Columbus, OH). Respiratory exchange ratio (RER) was calculated from VCO_2_ release-to-VO_2_ consumption ratio (VCO_2_ release/VO_2_ consumption). Physical activity of mice in the metabolic cages was measured by recording movements along the X-, Y- and Z-axes using infrared sensors^25^. BMR was corrected for both body weight and lean body mass obtained from body composition analysis.

### Oral Glucose Tolerance test

For glucose tolerance test (OGTT), mice were fasted for six hours and were orally given 20% glucose solution (dose: 2 g/Kg body weight). Blood was drawn from tail vein at 0, 15, 30, 60 and 120 minutes after the glucose load and blood glucose was measured by commercially available glucose strips (Contour Next, Bayer).

### Plasma biochemical measurements and Pancreatic Lipase Activity

Plasma TG (Wako Chemicals), FFA (Abcam), total, HDL and VLDL/LDL cholesterol levels were measured using commercially available kits according to the manufacturer’s instructions (Abcam). Plasma insulin was measured by ELISA (EMD Millipore). Pancreatic lipase activity was measured in the pancreatic tissue homogenates according to the manufacturer’s protocol (Abcam #ab102524).

### Immunohistochemistry

Epididymal adipose tissue, brown adipose tissue, liver, small intestines (3 different regions from proximal 10 cm at regular intervals) were formalin-fixed overnight at 4 °C and embedded in paraffin. Five micron sections were taken and stained for haematoxylin & eosin (H&E) to study tissue morphology. Picrosirius red (PSR) staining was used to visualize fibrillary collagen as reported previously^26^. Adipocyte size was measured in H&E sections using ImageJ software (100 cells/mouse, n = 4/group). For Oil Red O (ORO) staining of tissue neutral lipids, liver and skeletal muscle were embedded in tissue freezing medium immediately after dissection and stored at −80 °C. Five micron sections were used for staining and processed according to the protocol described previously^27^. Liver sections were stained with ORO for 15 minutes whereas skeletal muscle was stained for an hour. Adipose tissue inflammation was determined by staining for macrophages (F4/80 antibodies; AbD Serotec #MCA). Pancreas and small intestine sections were stained for TIMP4 (Abcam #ab51207) and also for pancreatic lipase (Santacruz #sc-393085) and CD36 (Novus Biologicals #NB400–144) respectively.

### Western blotting

Tissues were homogenized in ice-cold sigma buffer (Sigma Aldrich #C3228) with protease and phosphatase inhibitor cocktails. Enterocytes were harvested from the proximal segment of the small intestine (proximal 10 cm) after the intestine was rinsed with cold PBS to remove luminal contents. Homogenates were centrifuged at 1000 × *g* for 15 minutes at 4 °C and supernatants were collected. Protein concentration was determined by DC protein assay kit (Bio-Rad #5000116) and 40 μg of protein were resolved by electrophoresis on SDS-8% polyacrylamide gels. Proteins were transferred to PVDF membranes and were detected by the following primary antibodies: TIMP4 (Abcam #ab51207) and CD36 (Novus Biologicals #NB400-144). Respective HRP-conjugated secondary antibodies were used to detect immunoreactivity. Immunoreactive proteins were visualized using the ECL kit and band intensities were quantified by ImageJ software. Coomassie blue-stained gel was used as internal loading controls.

### mRNA expression analyses

RNA was extracted from adipose tissues, liver and enterocytes using TRIzol reagent (Life technologies). cDNA expression of the following genes was measured as described previously using Taqman RT-PCR (Thermo Fisher Scientic & Applied Biosystems)^28^; *Timp1*, *Timp2*, *Timp3*, *Timp4*, *Tnfα*, *Il-6*, *Il-1β*, *Mcp1*, *Ucp1*, *Scd1*, *Pparα*, *Elvol3*, *Col1α1*, *Col3α1*. Primer probe sequences are presented in supplementary table 1. Expression of 18s*rRna* gene was used as internal control.

### Hepatic, skeletal muscle and fecal lipid quantifications and Thin Layer Chromatography (TLC)

Liver and quadriceps muscle were homogenized in PBS and lipids were extracted by modified Folch’s method^25^. TG and cholesterol were measured using the commercially available kits (Wako and Abcam, respectively). Feces were collected after 24 hours and dried by lyophilizing overnight. For fecal TG analysis, total lipid was extracted and quantified as described above. TLC analysis was carried according to the protocol described previously using 100 mg of dried feces^30^.

### Statistics

Data are presented as dot-plot as well as averaged data as mean ± S.E.M. Statistical analyses were performed using two-way ANOVA followed by Tukey’s post-hoc test, or student t-test as indicated. Statistical significance was recognized at *P* < 0.05.

## Results

### Timp4-deficient mice resist high fat diet-induced visceral obesity

**Figure 1:**
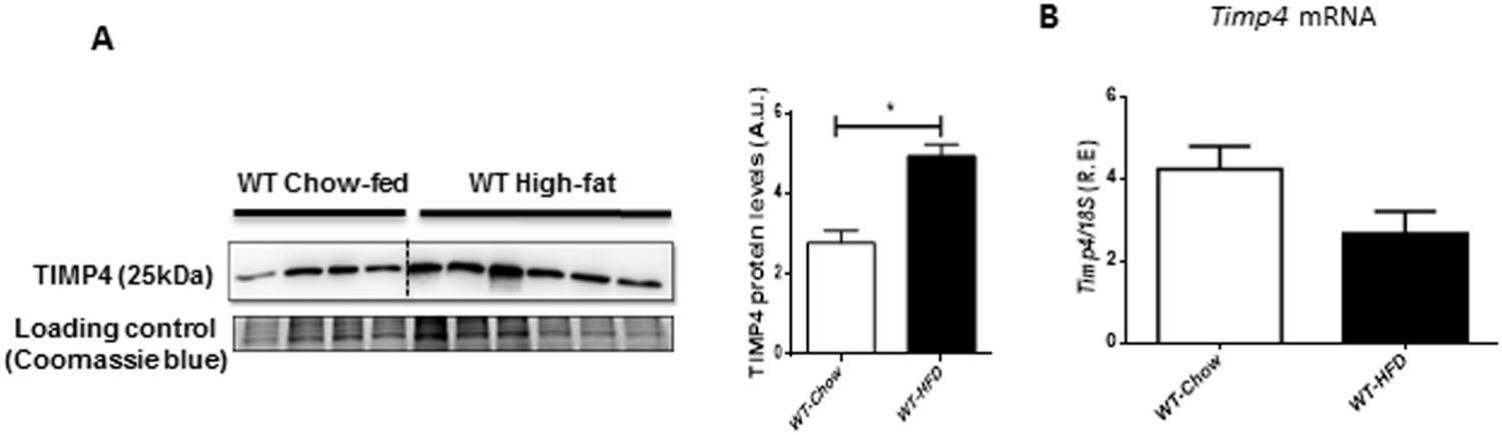
High fat feeding increased TIMP4 protein levels in adipose tissue. (**A**) Representative Western blot and averaged protein quantification for TIMP4 in epididymal fat pads in WT mice following chow or high fat diet (HFD). Coomassie blue-stained gel is used as the loading control. (**B**) *Timp4* mRNA expression in WT-chow and WT-HFD. 18S rRNA was used as internal control. n = 4–6. Averaged data are presented as mean ± S.E.M and analyzed by student’s t-test analysis. * indicates significance (p ≤ 0.05).

**Figure 2:**
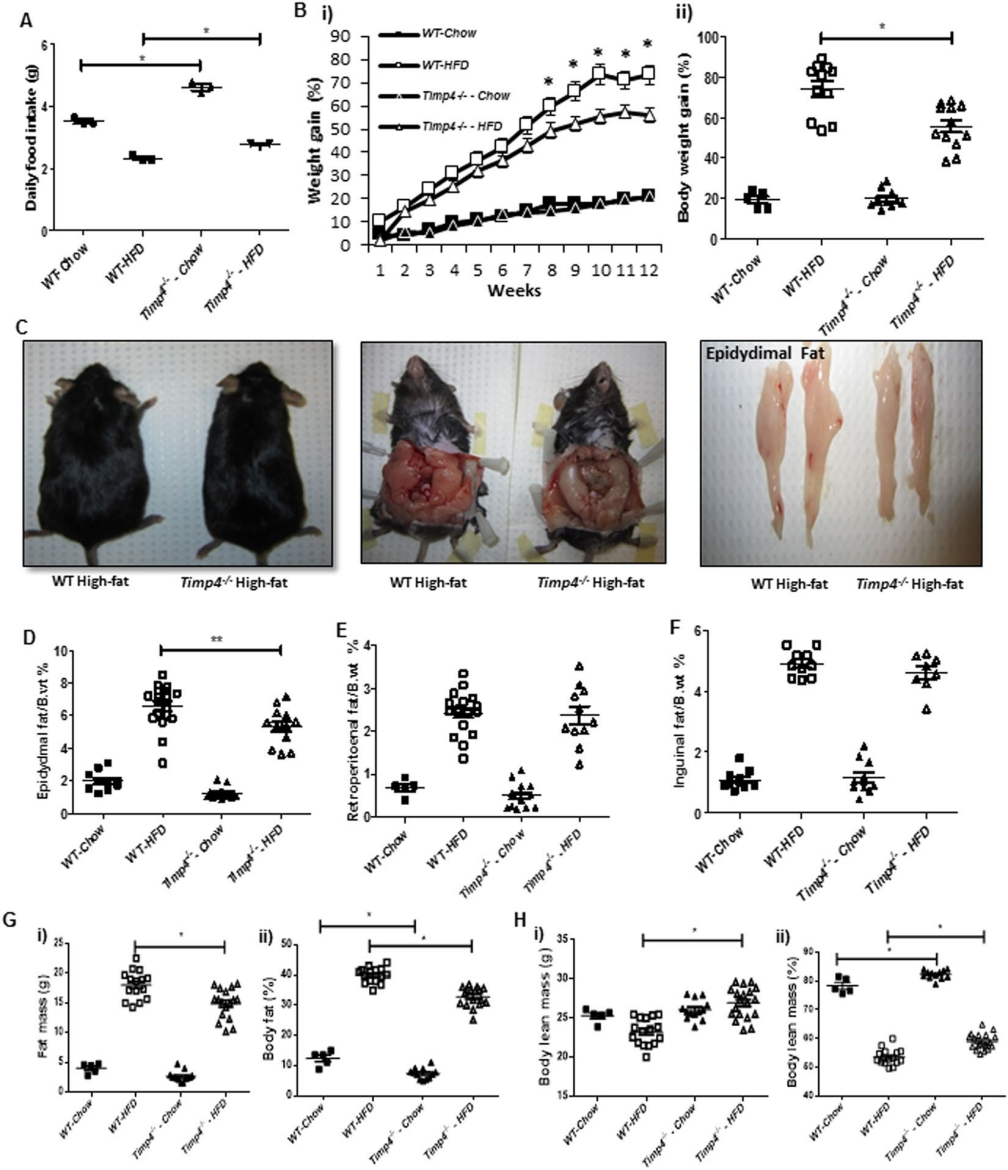
*Timp4* ^−/−^ mice exhibit increased food intake but reduced body fat percentage. Weekly food intake (**A**), rate of weekly body weight gain (**Bi**) and percent weight gain (**Bii**) in WT and T*imp4* ^−/−^ mice following chow or high fat diet (HFD). (**C**) Dorsal and ventral view, and macroscopic images of epididymal fat pads from high fat-fed WT and *Timp4* ^−/−^ mice. (**D**–**F**) Epidydimal fat, retroperitoneal fat and inguinal fat as percentage of body weight in indicated groups. (**G**–**H**) Whole body fat content (absolute, Gi; as percentage of body weight, **Gii**), and lean body mass (absolute, **Hi**; and as percentage of body weight, **Hii**) assessed by Echo-MRI body mass composition analysis. Significance between the chow-fed and HFD-fed groups within the genotypes are not shown in the figure. Averaged data are presented as mean ± S.E.M and analyzed by ANOVA. *p < 0.05 for the indicated groups.

TIMP4 protein levels increased significantly (by 77%) in the epidydimal adipose tissue of high fat diet-fed WT mice (WT-HFD) mice (Fig. 1A), although *Timp4* mRNA expression was not altered significantly (Fig. 1B). To investigate whether the increase in TIMP4 contributes to adipose tissue fat deposition and obesity, we fed WT and age-matched *Timp4* ^−/−^ mice HFD for 12 weeks. Prior to the start of HFD protocol, *Timp4* ^−/−^ mice exhibited significantly greater food intake (Fig. 2A), but a significantly higher body weight compared to the age-matched WT mice (Supplementary Figure 1), primarily due to a greater lean mass but lower fat mass (Fig. 2G). During the 12 weeks of HFD or chow feeding, chow-fed mice of both genotypes showed a similar rate of body weight gain, but *Timp4* ^−/−^ mice receiving HFD showed a reduced rate of weight gain compared to WT-HFD mice. Consistent with the leaner appearance of *Timp4* ^−/−^-HFD mice (Fig. 2B), the size of the visceral (epidydimal) fat pads were significantly reduced in these mice compared to WT-HFD (Fig. 2C,D), but no difference was observed in retroperitoneal (Fig. 2E) or inguinal fat pad size (Fig. 2F, Supplementary Figure 3) between the two HFD groups. We performed Echo-MRI to determine the body mass composition. Chow-fed *Timp4* ^−/−^ mice showed a significantly lower body fat percentage compared to chow-fed WT mice (Fig. 2Gii). Consistently, HFD-fed *Timp4* ^−/−^ mice exhibited a significantly lower body fat content (Fig. 2Gi) which comprised a lower percentage of total body weight (Fig. 2Gii), but a higher lean mass content (Fig. 2Hi) that comprised a higher percentage of the total body weight (Fig. 2Hii) compared to parallel WT groups (Fig. 2G). These results indicate that *Timp4* gene deletion was associated with decreased body fat accumulation despite increased food intake.

### Timp4-deficiency reduced white adipose tissue hypertrophy and reduced inflammation

**Figure 3:**
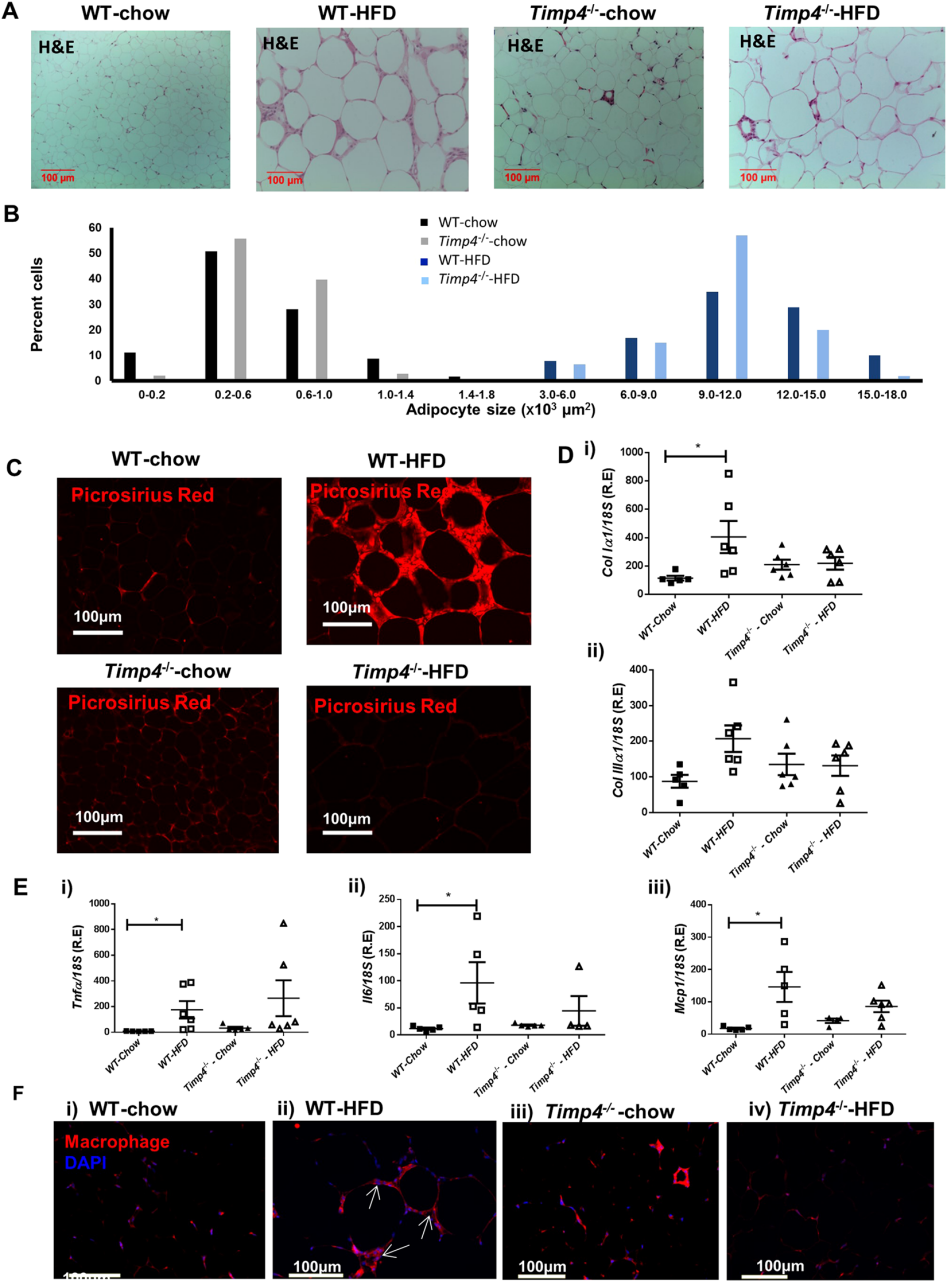
Reduced adipocyte size, fibrosis and inflammation in *Timp4* ^−/−^ mice on high fat diet. H&E staining (**A**) and adipocyte size distribution (**B**) in epidydimal adipose tissue of indicated groups. Picrosirius Red staining (**C**), and mRNA expression of collagen 1α1 (*Col1α1*)(Di) and collagen IIIα1 (*ColIIIα1*) (Dii). (**E**) mRNA expression of *Tnfα* (i), *Il6* (ii), and *Mcp1* (iii). (**F**) Immunostaining to detect macrophages in epidydimal adipose tissue (F4/80 antibody). Averaged data are presented as mean ± S.E.M and analyzed by ANOVA. *Indicates significance (p < 0.05).

To gain more mechanistic insight, we next examined the impact of *Timp4*-deficiency on epidydimal adipose tissue morphology. Despite the significant decrease in body fat percentage in chow-fed *Timp4* ^−/−^ mice, these mice showed a higher percentage of large-sized adipocytes in the epidydimal fat compared to chow-fed WT mice (Fig. 3A,B). This could possibly result from impaired pre-adipocyte recruitment or differentiation in chow-fed *Timp4* ^−/−^ mice leading to TG accumulation in the existing adipocytes rather than generation of new adipocytes. In contrast to chow-fed mice, the lower body fat content in HFD-fed *Timp4* ^−/−^ mice was associated with smaller adipocyte size when compared to that of HFD-fed WT mice (Fig. 3A and B).

Increased collagen fiber deposition (Fig. 3C) and collagen type I expression (Fig. 3D) were observed in WT-HFD but not in *Timp4* ^−/−^-HFD adipose tissue. In addition, expression of inflammatory cytokines *Tnfα*, *Il-6* and *Mcp1* were elevated in WT-HFD but not in *Timp4* ^−/−^-HFD adipose tissue (Fig. 3Ei–iii). Further, macrophage infiltration was increased only in WT-HFD epidydimal fat evident from the crown-like appearance of adipocytes (Fig. 3Fi–iiii). The HFD-induced rise in expression of inflammatory markers did not reach statistical significance in *Timp4* ^−/−^ mice, while macrophage infiltration was markedly lower in these mice compared to WT-HFD mice (Fig. 3F). *Timp4* ablation did not alter the expression of other *Timp* genes and genes related to lipid metabolism (*Ucp1*, *Elovl3*, *Acc1α*, *Dgat1* and *Srebp1*) in the epidydimal adipose tissue of chow-fed or HFD-fed *Timp4* ^−/−^ mice, but significantly increased *Dgat2* in *Timp4* ^−/−^-HFD adipose tissue (Supplementary Figure 2). These results indicate that *Timp4* deficiency suppresses hypertrophy, fibrosis and inflammation in epidydimal adipose tissue upon HFD-feeding.

### Obesity-induced hepatic steatosis was suppressed in Timp4-deficient mice

**Figure 4:**
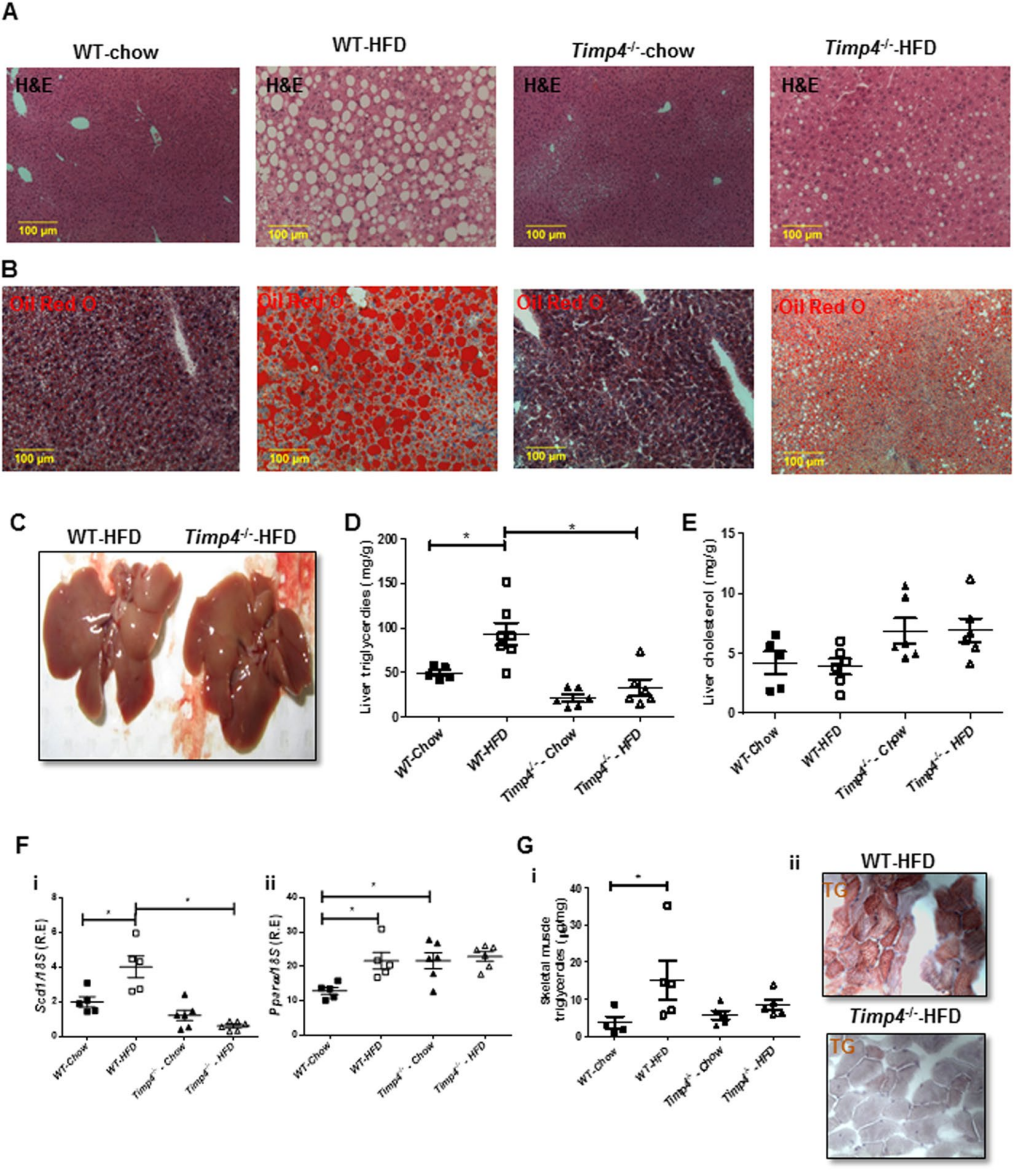
*Timp4* gene deletion protects mice from HFD-induced hepatic steatosis. H and E staining (**A**) and Oil Red-O staining (**B**) of fixed liver after 12 -weeks of chow on high fat diet (HFD) in the indicated genotypes. Representative picture of livers WT-HFD and *Timp4* ^−/−^HFD mice (**C**). Liver TG content (**D**) and hepatic cholesterol content (**E**) in indicated groups. (**F**) mRNA expression of *Scd1* (i) and *Pparα* (ii) in the liver. (**G**) Skeletal muscle TG content (i) and fat deposition as indicated by Oil Red-O staining (ii) (400X). Averaged data are presented as mean ± S.E.M and analyzed by ANOVA. *Indicates significance (p < 0.05).

Although *Timp4* gene expression is negligible in the liver (data not shown), because altered adipose tissue function can significantly affect the hepatic function, we investigated the hepatic lipid content, histology and expression of genes related to lipid metabolism. The chow-fed mice of either genotype showed comparable hepatic cellular morphology (Fig. 4A), whereas hepatic lipid content was significantly reduced in *Timp4* ^−/−^-HFD compared to WT-HFD mice as assessed by H&E (Fig. 4A), Oil Red-O staining (Fig. 4B) and the macroscopic appearance of the liver that appears more white in WT-HFD but not in *Timp4* ^−/−^-HFD (Fig. 4C). Liver TG was significantly elevated in WT-HFD but not in *Timp4* ^−/−^-HFD mice compared to the corresponding chow-fed groups (Fig. 4D), while hepatic cholesterol levels were comparable among all groups (Fig. 4E). HFD also induced expression of lipogenic (*Scd1*) and lipid oxidation (*Pparα*) genes, in WT but not in *Timp4* ^−/−^ mice (Fig. 4Fi-ii). Hepatic fibrosis was not detected in any group (Supplementary Figure 4). Expression of *Timp3*, but not *Timp1* or *Timp2*, was elevated in chow-fed *Timp4* ^−/−^ mice, although the expression of all three *Timp* mRNAs was comparable between HFD-fed mice of either genotype (Supplementary Figure 4). Along with reduced TG levels, skeletal muscle TG content was also markedly lower in *Timp4* ^−/−^-HFD compared to WT-HFD mice (Fig. 4G).

### HFD-mediated induction of plasma cholesterol, fatty acids and hyperinsulinemia were suppressed in Timp4-deficient mice

**Figure 5:**
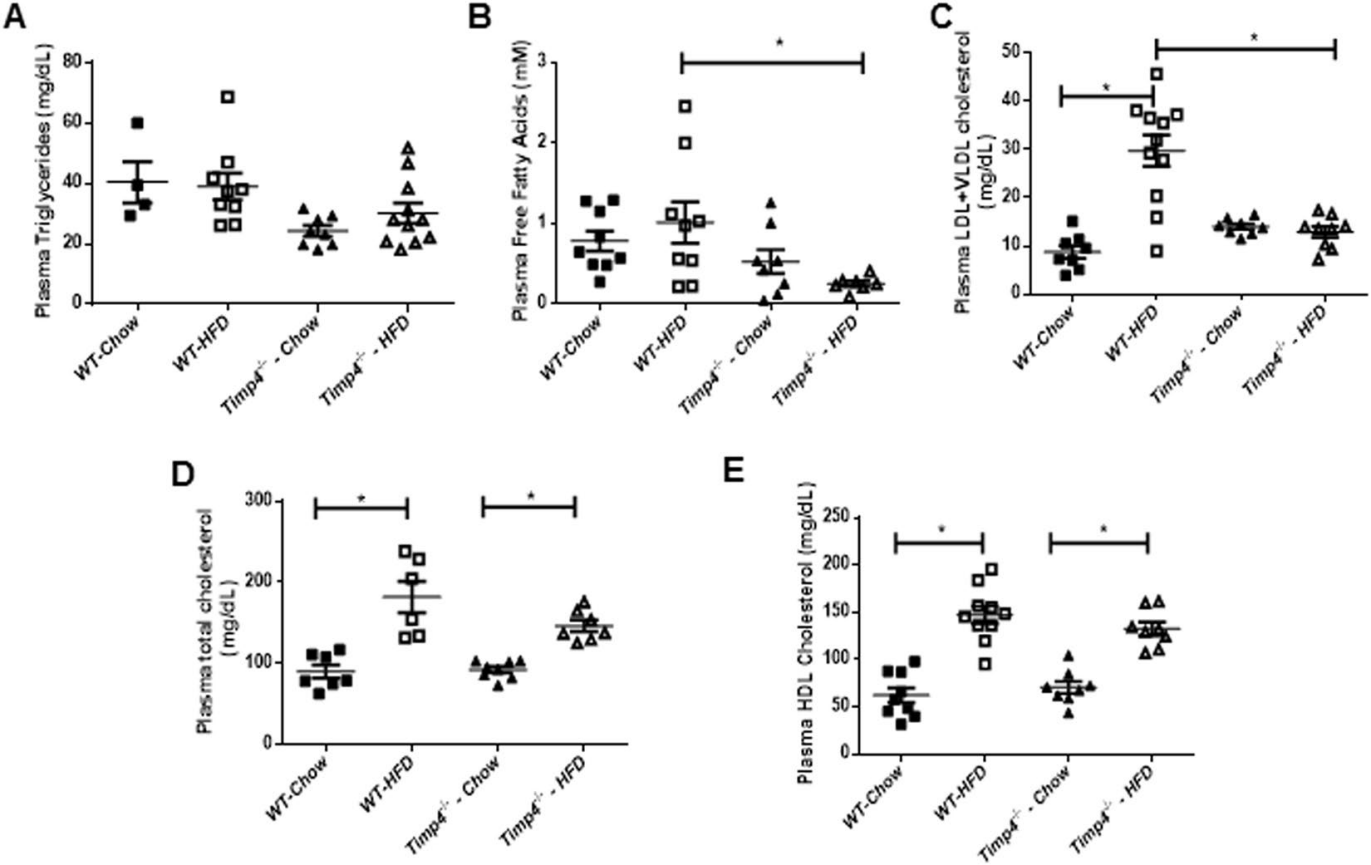
*Timp4* ^−/−^ mice show protection against HFD-induced elevations in plasma free fatty acids and LDL-cholesterol. Fasting plasma triglyceride (TG) (**A**), free fatty acids (**B**), LDL and VLDL cholesterol (**C**), total cholesterol (**D**) and HDL cholesterol levels (**E**) in indicated groups. Averaged data are presented as mean ± S.E.M and analyzed by ANOVA. * indicates significance (p < 0.05).

Plasma TG levels were comparable among all groups (Fig. 5A), whereas the HFD-induced rise in free fatty acid and total LDL-cholesterol levels (LDL + VLDL) were significantly blocked in *Timp4* ^−/−^ compared to WT mice (Fig. 5B,C). Plasma total cholesterol and HDL cholesterol similarly increased with HFD in both genotypes (Fig. 5D,E).

**Figure 6:**
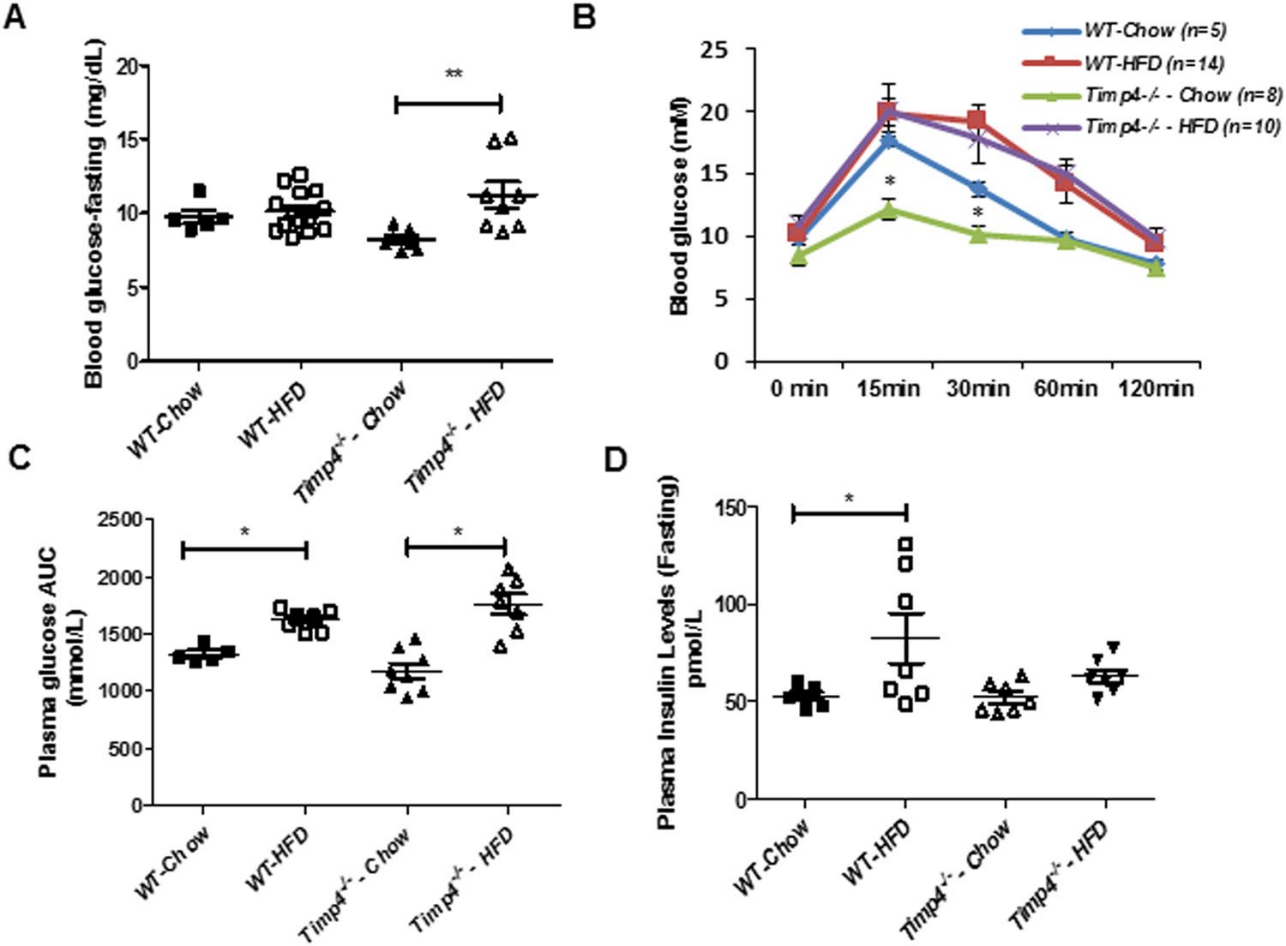
*Timp4* gene deletion protects mice from HFD-induced hyperinsulinemia but not from glucose intolerance. Fasting plasma glucose levels (**A**), blood glucose levels during an oral glucose tolerance test (OGTT) (**B**), and the averaged area under curve (AUC) for indicated groups (**C**). (**D**) Fasting plasma insulin levels. Averaged data are presented as mean ± S.E. and analyzed by ANOVA. * indicates significance (p < 0.05) between WT chow-fed and TIMP4KO chow-fed groups.

Since *Timp4* ^−/−^ HFD mice showed reduced adiposity and plasma FFAs, we investigated if this led to improved obesity-associated glucose intolerance. Fasting glucose levels were comparable between the chow-fed and the HFD-fed groups, however, the HFD-induced rise in blood glucose was greater in *Timp4* ^−/−^ mice (Fig. 6A). Oral glucose tolerance test revealed that chow-fed *Timp4* ^−/−^ mice have significantly lower glucose levels at 30 minutes and 60 minutes compared to WT-chow mice (Fig. 6B) although the difference in total glucose AUC (area under the curve) did not reach statistical significance (Fig. 6C). However, after 12 weeks of HFD, *Timp4* ^−/−^ and WT mice exhibited the same degree of glucose intolerance (Fig. 6B,C) indicating that the reduced adiposity in *Timp4* ^−/−^-HFD mice did not exert protection against glucose intolerance. Plasma insulin levels were similar in chow-fed WT and *Timp4* ^−/−^ mice, while the high fat-induced rise in insulin was only detected in WT but not in *Timp4* ^−/−^ mice (Fig. 6D).

### Brown Adipose Tissue (BAT) content was unaltered in Timp*4*^−/−^ mice

The reduced body fat gain despite increased food intake in *Timp4* ^−/−^ mice could be due to increased energy expenditure and enhanced BAT thermogenesis. We assessed the histomorphometery of BAT tissue as well as expression of genes encoding regulatory proteins of thermogenesis (*Ucp1*) and brown adipose tissue fat accretion (*Elovl3*) in the HFD-fed mice. We found less accumulation of fat in *Timp4* ^−/−^ BAT compared to the corresponding WT (assessed by the smaller size of adipocytes), a similar pattern to what we observed in white adipose tissue (Supplementary Figure 5A). *Ucp1* expression was comparable among all groups (Supplementary Figure 4Bi). *Elovl3* mRNA expression were similar between WT-HFD and *Timp4* ^−/−^-HFD mice, although its HFD-induced rise was significantly greater in *Timp4* ^−/−^ mice (Supplementary Figure 5Bii). Consistent with these observations, body temperature in HFD-fed *Timp4* ^−/−^ mice was similar to WT-HFD mice (36.6 ± 0.21 °C vs 36.3 ± 0.27 °C).

### Timp*4*^−/−^ mice have reduced basic metabolic rate (BMR), energy expenditure (EE) and respiratory exchange ratio (RER)

**Figure 7:**
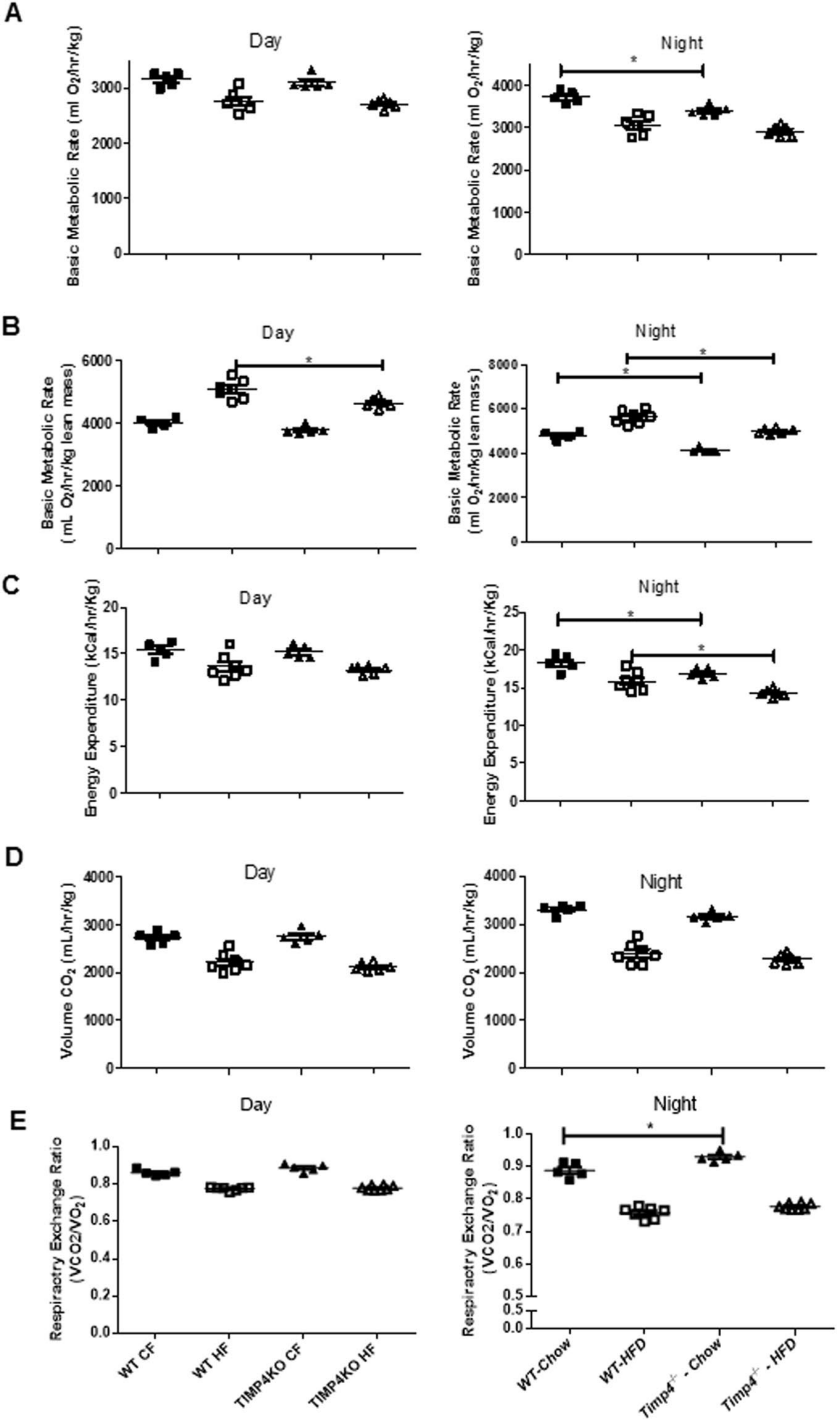
Reduced basic metabolic rate, energy expenditure and increased respiratory exchange ratio in *Timp4* ^−/−^ mice. Basic metabolic rate corrected for body weight (BMR)(**A**), basic metabolic rate corrected for lean mass (**B**), energy expenditure (**C**), volume of released CO_2_ (**D**), and respiratory exchange ratio (RER) (**E**) during day (i) and nigh time (ii) in chow- or HFD-fed WT and *Timp4* ^−/−^ mice. Significance between the chow-fed and HFD-fed groups within the genotypes are not shown in the figure. Averaged data are presented as mean ± S.E.M and analyzed by ANOVA. * indicates significance (p < 0.05).

Since BAT analysis did not reveal increased thermogenesis in *Timp4* ^−/−^ mice, we investigated whether the basic metabolic rate (BMR) and the related parameters were altered in these mice. In contrary to our anticipation, *Timp4* ^−/−^chow fed mice showed significantly lower BMR compared to WT-chow-fed mice during night when data were normalized to whole body mass (Fig. 7A). When BMR was normalized to lean mass, *Timp4* ^−/−^-HFD mice further showed reduced BMR at day and night, as well as the observed difference between chow-fed groups (Fig. 7B). This could be due to the varying body fat content in HFD groups which resulted in different BMR values when BMR was normalized to whole body weight whereas to lean body mass. Consistently, night time energy expenditure (EE) was significantly lower in chow and high fat-fed *Timp4* ^−/−^ compared to the corresponding WT mice (Fig. 7C). Interestingly, while volume CO_2_ was similar between the two genotypes (Fig. 7D), respiratory exchange rate was greater in *Timp4* ^−/−^chow compared to WT-chow mice, but not different between the HFD groups (Fig. 7E). Chow-fed and HFD-fed *Timp4* ^−/−^ showed similar physical activity (day and night) compared to the respective WT mice (Supplementary Figure 6).

### Timp*4*^−/−^ mice have defective lipid absorption and reduced CD36 protein levels

**Figure 8:**
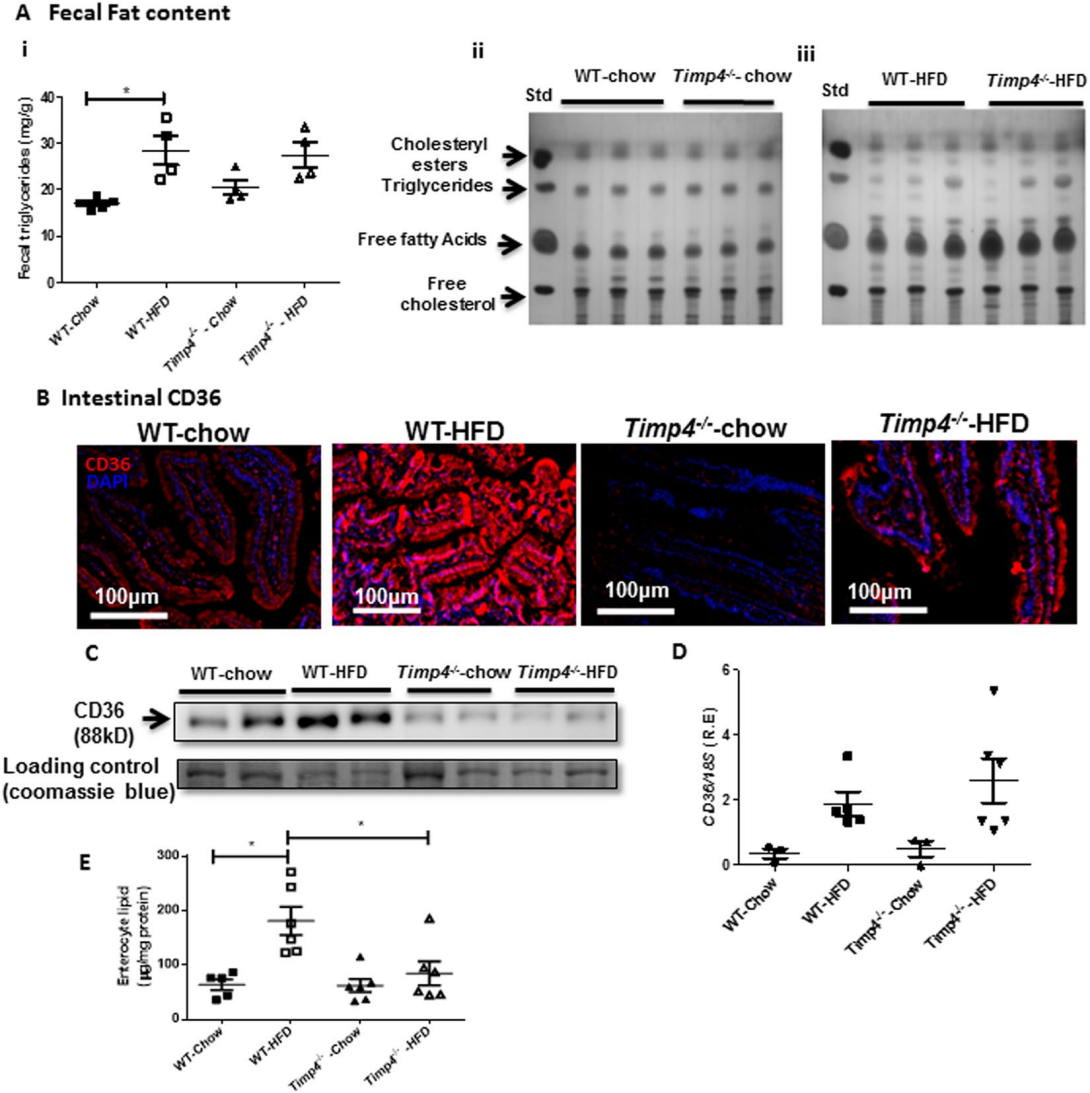
*Timp4*-deficiency results in defective lipid digestion and absorption. Fecal triglyceride (TG) content (**Ai**) in chow- or HFD-fed WT and *Timp4* ^−/−^ mice. Fecal lipid analysis by thin layer chromatography in chow-fed (**Aii**) and HFD-fed (iii) WT and *Timp4* ^−/−^ mice. (**B**) Immunostaining for CD36 in small intestine (proximal region) of chow-fed and HFD-fed WT and *Timp4* ^−/−^ mice. Western blot (**C**) and mRNA (**D**) levels for CD36 in enterocyte fraction of chow-fed and HFD-fed WT and *Timp4* ^−/−^ mice (collected from the proximal small intestine). (**E**) Triglyceride levels in enterocytes collected from the proximal small intestine. Averaged data are presented as mean ± S.E.M and analyzed by ANOVA. * indicates significance (p < 0.05).

Increased food intake along with decreased BMR should lead to increased body fat and severe obesity, but in contrast, *Timp4* ^−/−^ mice exhibit decreased body fat and reduced obesity following HFD feeding. This suggests that lipid absorption and/or digestion could be impaired in *Timp4* ^−/−^ mice. Fecal TG levels showed increased trend in the *Timp4* ^−/−^ chow-fed mice when compared to WT-chow-fed mice (Fig. 8A), whereas fecal TG levels were significantly increased in WT-HFD but not in *Timp4* ^−/−^-HFD compared to corresponding chow-fed groups (Fig. 8A). Further analysis of fecal lipid profile by TLC demonstrated similar lipid contents between chow-fed WT and *Timp4* ^−/−^ mice (Fig. 8Aii). Interestingly, *Timp4* ^−/−^-HFD mice showed a striking increase in fecal free fatty acid levels with unaltered TG, cholesteryl esters, and free cholesterol levels compared to WT-HFD group (Fig. 8Aiiii). These data suggest that when exposed to high fat diet, *Timp4*-deficiency is associated with suppressed fatty acid absorption.

CD36 is a major fatty acid transporter, and since we found reduced fatty acid absorption in *Timp4* ^−/−^ mice, we investigated if CD36 levels are altered in *Timp4*-deficient mice. We assessed the expression of CD36 in the proximal small intestine (duodenum) by immunofluorescent staining (Fig. 8B) and immunoblotting (Fig. 8C) and found that CD36 protein levels are significantly lower in enterocytes of chow-fed *Timp4* ^−/−^ compared to WT mice, and the marked HFD-induced rise in CD36 levels observed in WT mice was not detected in *Timp4* ^−/−^ mice. *Cd36* mRNA, on the other hand, is increased similarly in the enterocytes of the proximal intestine following HFD in both genotypes (Fig. 8D). Moreover, lipid content in enterocytes from *Timp4* ^−/−^-HFD mice was significantly lower than that in WT-HFD (Fig. 8E). Interestingly, TIMP4 is expressed in the enterocyte of small intestine and its protein and mRNA levels are elevated with high-fat diet in WT mice (Supplementary Figure 7A,B). These data support the notion that TIMP4 could be responsible for post-translational regulation of CD36 and fat absorption in the small intestine.

We further assessed whether *Timp4*-deficiency alters fat digestion since TIMP4 is also expressed across the pancreas of WT mice and its levels are increased with high fat diet (Supplementary Figure 7C). Examining pancreatic lipase levels and activity revealed that the HFD-induced rise in lipase levels were similar in both genotypes (Supplementary Figure 7D), while HFD induced a rise in lipase activity in WT but not in *Timp4* ^−/−^ mice, although the difference between WT-HFD and *Timp4* ^−/−^-HFD mice did not reach statistical significance (Supplementary Figure 7D).

## Discussion

This study is the first to report the role of TIMP4 in high-fat diet-induced obesity. *Timp4* gene deletion suppressed HFD-induced weight gain despite increased food intake and decreased BMR. *Timp4* ^−/−^ mice fed HFD exhibit defective lipid absorption leading to reduced adipocyte hypertrophy, fibrosis, and diminished hepatic steatosis and dyslipidemia. Suppressed lipid absorption in *Timp4* ^−/−^ mice is associated with reduced CD36 protein levels in the small intestine and reduced lipid content in the enterocytes. CD36 is a membrane protein and acts as translocator of fatty acid and oxidized sterols across the cell membrane^31^. CD36 is expressed mostly in the proximal region of the small intestine and shown to be important for FA uptake as well as secretion and clearance of intestinal derived lipoproteins^32^. The decreased CD36 protein, but not its mRNA levels in the small intestine of *Timp4* ^−/−^ mice could be due to elevated MMP activities in the absence of MMP-inhibitory function of TIMP4, leading to cleavage of the membrane-bound CD36 in this region as has been reported for the heart and spleen^33, 34^, and as recently reported that CD36 can be cleaved by MMP9^34^.

Adipose tissues express various MMPs and TIMPs which have been reported to be dysregulated in obesity^12^. Inhibition of MMPs impairs adipose tissue development, decreases adipocyte hypertrophy and increases collagen deposition^35, 36^. TIMP1 has been linked to adiposity in humans, and recombinant TIMP1 inhibited preadipocyte differentiation and to decrease HFD-induced adipocyte hypertrophy^19, 37^, and TIMP1 gene deletion increased fecal caloric excretion^37^. Loss of *Timp2*, on the other hand, increased susceptibility to HFD-induced obesity^38^. *Timp3* gene deletion increased adipose tissue inflammation whereas macrophage overexpression of TIMP3 resulted in protection from inflammation^22, 23^. HFD induced a rise in TIMP4 protein but not its mRNA levels in the adipose tissue. This could be due to post-transcriptional regulation of TIMP4 protein or perhaps a greater stability (half-life) of TIMP4 protein compared to its mRNA. Decreased body fat accumulation in *Timp4* ^−/−^-HFD mice could be attributed to decreased systemic entry of lipids due to impaired lipid absorption. Although defective fat absorption appears to be the main mechanism underlying the observed phenotype, decreased insulin levels in HFD-fed *Timp4* ^−/−^ mice could also contribute to the decreased adipose tissue depots in these mice since insulin can induce hepatic lipogenesis, increase circulatory TG levels and TG uptake by the adipose tissue in addition to its lipogenic effect on adipose tissue. Decreased lipid digestion cannot be completely ruled out in HFD-fed *Timp4* ^−/−^ mice as these mice showed a strong trend in decreased pancreatic lipase activity compared to HFD-fed WT mice.

With respect to our observations in the *Timp4* ^−/−^chow fed mice, lack of TIMP4 can lead to increased extracellular matrix turnover and deposition, which can in turn limit adipose tissue expansion as reported previously^35, 36^ leading to an increase in the adipocyte size as we observed in chow-fed *Timp4* ^−/−^ these mice. These observations suggest that TIMP4 is important for the maintenance of adipose tissue homeostasis under normal conditions. *Timp4* ^−/−^chow fed mice showed similar features as the HFD-fed *Timp4* ^−/−^ mice in terms of increased food intake, decreased body fat and BMR, but they did not exhibit an overt defect in lipid digestion or absorption compared to WT-chow fed mice. However, the lower fecal TG and free fatty acid content, and enterocyte CD36 protein levels compared to WT-chow fed mice, although did not reach statistical significance, could underlie the enhanced susceptibility of *Timp4* ^−/−^ mice to compromised lipid absorption when exposed to high fat diet.

*Timp2* ^−/−^ male mice develop obesity-induced insulin resistance leading to diabetic state due to pancreatic β-cell exhaustion, whereas macrophage-specific TIMP3 expression protects from insulin resistance^21, 22^. We found that *Timp4* ^−/−^ chow-fed mice show lower plasma glucose levels (up to 60 minutes following glucose loading) despite normal insulin levels and also have lower glucose levels after glucose load at 30 and 60 mins. This could be due to increased systemic utilization of glucose secondary to lower availability of lipid stores for energy production. This is consistent with the increased RER in *Timp4* ^−/−^ chow-fed mice. *Timp1* ^−/−^ mice have unaltered BMR, whereas mice with macrophage-specific overexpression of TIMP3 showed a small increase in BMR^22, 37^. The decreased BMR in *Timp4* ^−/−^-HFD mice indicates that these mice have reduced energy expenditure, and that enhanced energy expenditure (possibly through increased BAT activity) is not the cause of their reduced adiposity. Interestingly, *Timp4* ^−/−^ HFD-fed mice are not protected from obesity-induced glucose intolerance despite their decreased body fat, plasma TG and fatty acids, which have been linked to glucose intolerance through impairing insulin signaling; however, *Timp4* ^−/−^-HFD mice are protected from hyperinsulinemia and accumulation of intramuscular TG.

MMP inhibitors have been shown to protect mice from HFD-induced fatty liver and shown to increase *Pparα* gene expression^39^. Hepatic TIMP1 and TIMP2 levels are elevated in obesity-related non-alcoholic fatty liver disease (NAFLD), and TIMP1 has been shown to mediate HFD-induced hepatic steatosis in mice^37^. Macrophage-specific overexpression of TIMP3 protected mice from hepatic steatosis^22, 23^. In contrast to the high expression levels of *Timp1*, *Timp2* and *Timp3* in liver, *Timp4* levels in liver is negligible, suggesting that it may not play a role in hepatic ECM turnover, and absence of hepatic fibrosis in *Timp4* ^−/−^ livers is likely secondary to the reduced fat absorption, and the subsequent suppression of adipocyte hypertrophy and inflammation. Hepatic TG levels are regulated by the rate of synthesis, secretion into plasma, in VLDL, and oxidation. *Timp4* ^−/−^ mice have reduced hepatic TG stores. Because plasma fatty acid levels were low in *Timp4* ^−/−^ mice particularly under high-fat conditions, hepatic esterification and secretion of TG would be expected to be decreased leading to the observed lower hepatic TG in these mice. Additionally, upregulation of *Pparα*, expression together with decreased expression of *Scd1* suggests plausible increase in fatty acid oxidation and decrease in fatty acid synthesis in *Timp4* ^−/−^-HFD mice, which might have contributed to the observed decrease in hepatic TG stores^40, 41^. The protective effects of *Timp4* gene deletion on hepatic steatosis could also be due to the decreased insulin levels in HFD-fed mice as insulin is a potent activator of hepatic lipogenesis. In addition, *Timp4* ^−/−^-HFD mice do not exhibit hypercholesterolemia mainly due to lower LDL-cholesterol levels, suggesting a possible role of TIMP4 in cholesterol metabolism. Decreased VLDL secretion may be responsible for the observed decrease in the LDL-cholesterol levels as VLDL particles transform to LDL particles upon TG delivery to the peripheral tissues.

In summary, our study demonstrates that absence of TIMP4 suppresses HFD-induced obesity possibly by impairing lipid absorption. This can further hinder the complications associated with obesity including hepatic steatosis, dyslipidemia, as well as adipocyte hypertrophy, inflammation, and fibrosis. Considering the multiple protective effects of *Timp4* gene deletion against obesity and associated comorbidities, targeted inhibition of TIMP4 could be a promising therapeutic target against obesity.

## Electronic supplementary material

Supplementary Information
