# Supplementary material for: Absence of Tissue Inhibitor of Metalloproteinase-4 (TIMP4) ameliorates high fat diet-induced obesity in mice due to defective lipid absorption
Source: Sci Rep. 2017 Jul 24;7:6210. doi: 10.1038/s41598-017-05951-4 (PMC5524827; doi:10.1038/s41598-017-05951-4)
Supplement: Supplementary file 1 — Supplementary Information [file 41598_2017_5951_MOESM1_ESM.pdf]

SUPPLEMENTARY TABLE AND FIGURES

Absence of Tissue Inhibitor of Metalloproteinase-4 (TIMP4) ameliorates high fat diet-induced obesity in mice due to defective lipid absorption

Siva S.V.P Sakamuri[1], Russell Watts[3], Abhijit Takawale[1], Xiuhua Wang[1], Samuel Hernandez-Anzaldo[2], Wesam Bahitham[3], Carlos Fernandez-Patron[2], Richard Lehner[3], Zamaneh Kassiri [1, *]

[1] Department of Physiology, Faculty of Medicine and Dentistry, University of Alberta, Edmonton, AB
[2] Department of Biochemistry, Faculty of Medicine and Dentistry, University of Alberta, Edmonton, Alberta, Canada
[3] Group on Molecular and Cell Biology of Lipids, Faculty of Medicine and Dentistry, University of Alberta, Edmonton, Alberta, Canada

Gene	Primer/Probe Sequences
Timp1	Forward: 5'-GACGGCCTTCTGCAATTCC-3'<br>Reverse: 5'-GTATAAGGTGGTCTGGTTGACTTCTG-3'<br>Probe: 5'-FAM-ACCTCGTCATCAGGGCCAAGTTCGT-TAMRA-3'
Timp2	Forward: 5'-GAGCCTGAACCACAGGTACCA-3'<br>Reverse: 5'-AGGAGATGTAGCACGGGATCA-3'<br>Probe: 5'-FAM-CTGCGAGTGCAAGATCACGCGC-TAMRA-3'
Timp3	Forward: 5'-CCAGGACGCCTTCTGCAA-3'<br>Reverse: 5'-CCCCTCCTTTACCAGCTTCTTC-3'<br>Probe: 5'-FAM-CGACATCGTGATCCGGGCCA-TAMRA-3'
Timp4	Forward: 5'-CACCCTCAGCAGCACATCTG-3'<br>Reverse: 5'-GGCCGGAACTACCTTCTCACT-3'<br>Probe: 5'-FAM-CACTCGGCACTTGTGATTCGGGC-TAMRA-3'
	**Assay ID**
Il1β	Mm00434228_m1
Il6	Mm00446190_m1
Mcp1	Mm00441242_m1
Tnfα	Mm00443258_m1
Acaca	Mm01304257_m1
Acs	Mm00484217_m1
Cd36	Mm00450236_m1
Col Iα1	Mm00801666_g1
Col IIIα1	Mm01254476_m1
Dgat1	Mm00515643_m1
Dgat2	Mm00499536_m1
Elovl3	Mm00468164_m1
NADH dehydrogenas	Mm001329746_m1
Pparα	Mm00440939_m1
Scd1	Mm00772290_m1
Srebp1	Mm00550338_m1

Supplementary Table 1 –The sequence for custom-designed primers and probes, and the assay ID for the pre-mixed primer/probe.

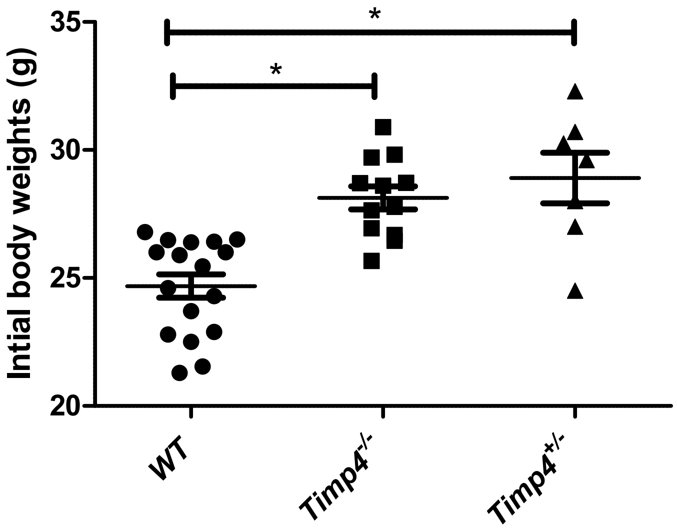

Supplementary Figure 1: ***Timp4*-/- mice have increased body weight.** Body weights of 8 week-old wild type (WT), *Timp4*$^{-/-}$ and TIMP4 heterozygous (*Timp4*$^{+/-}$) mice. Data are represented as Mean ± S.E and analyzed by ANOVA. * indicates significance ($p<0.05$).

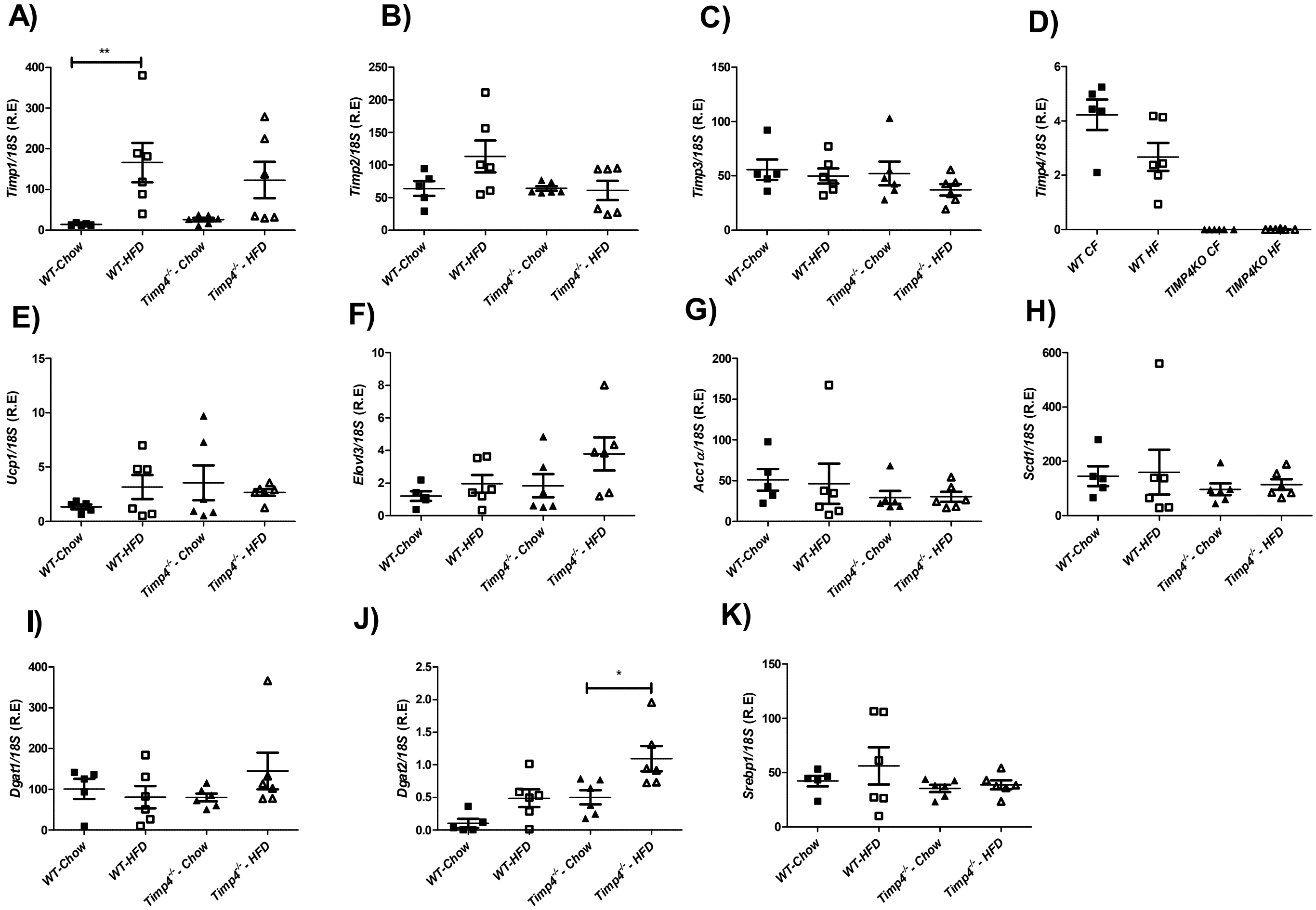

Supplementary Figure 2R1: *Timp4* gene deletion not altered expression of other *Timp*s or lipid metabolism genes in epidydimal adipose tissue. Relative gene expression of *Timp1* (A), *Timp2* (B), *Timp3* (C), *Timp4* (D), *Ucp1* (E), *Elovl3* (F), *Accα* (G), *Scd1* (H), *Dgat1* (I), *Dgat2* (J), *Srebp1* (K). mRNA levels were normalized by 18s rRNA levels (as internal control). Data are represented as Mean ± S.E and analyzed by ANOVA. * indicates significance (p≤0.05).

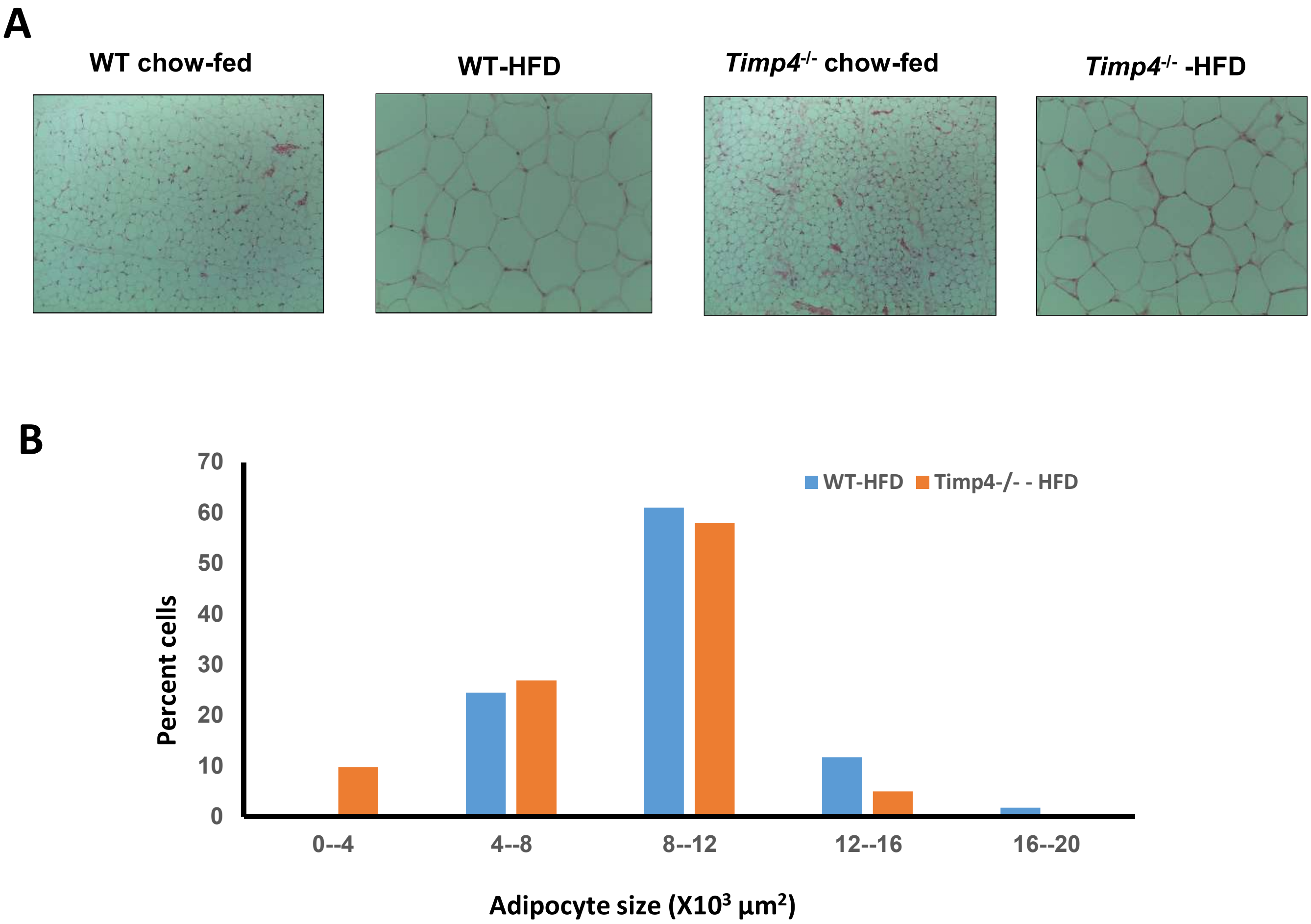

Supplementary Figure 3R1: (A) H&E staining of subcutaneous inguinal fat pad in the indicated groups.(B) Distribution of adipocyte size in subcutaneous inguinal fat pad in the indicate groups. HFD=high fat diet.

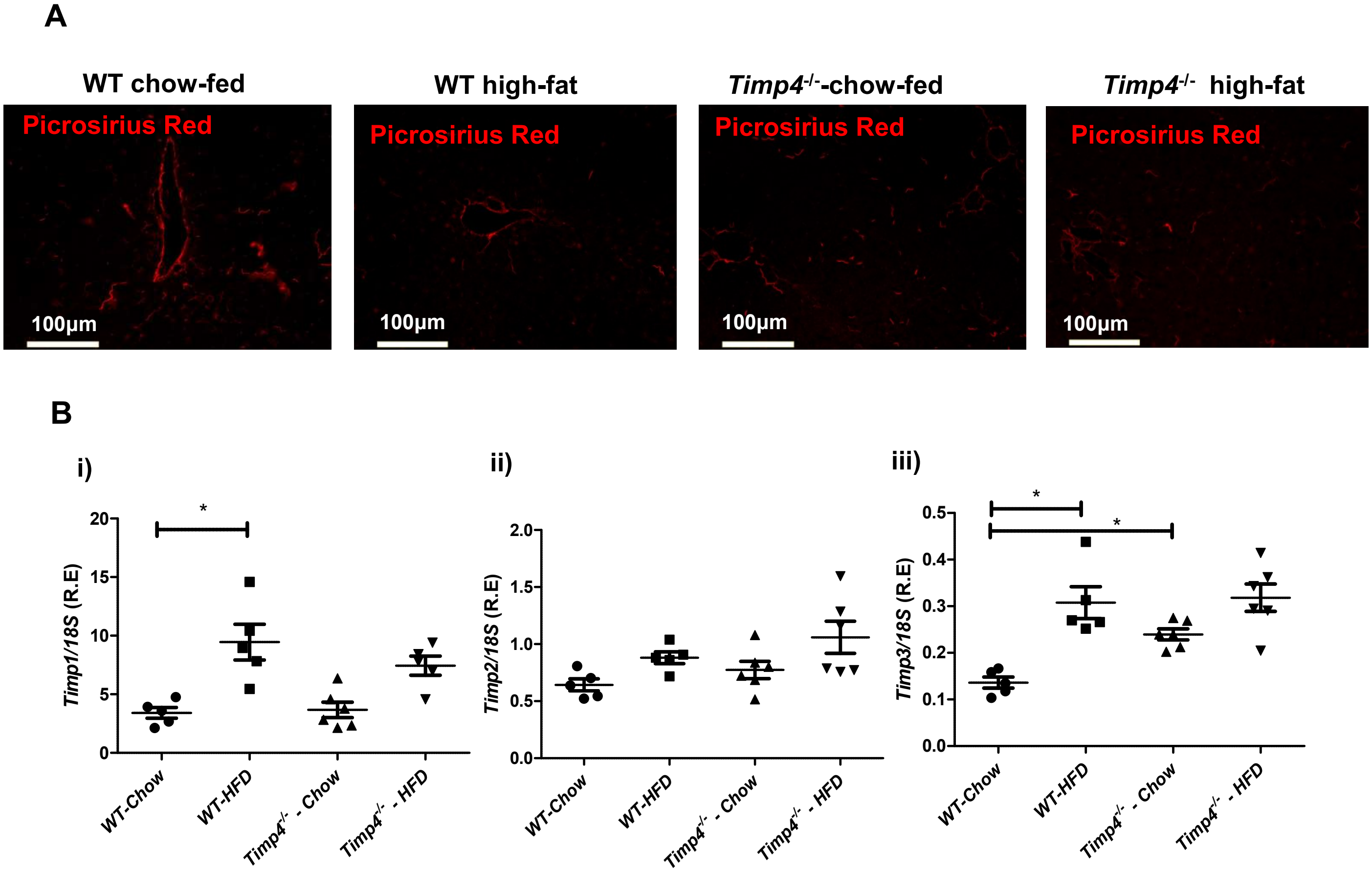

Supplementary Figure 4: *Timp4* gene deletion not altered hepatic ECM remodeling but induced *Timp3* expression in chow-fed mice. (A). Picrosirius staining. (B) Relative gene expression of ***Timp***1 (i), ***Timp***2 (ii), ***Timp***3 (iii). Gene expression of target genes was normalized by using 18s rRNA expression as internal. Data are represented as Mean ± S.E and analyzed by ANOVA. * indicates significance ($p<0.05$).

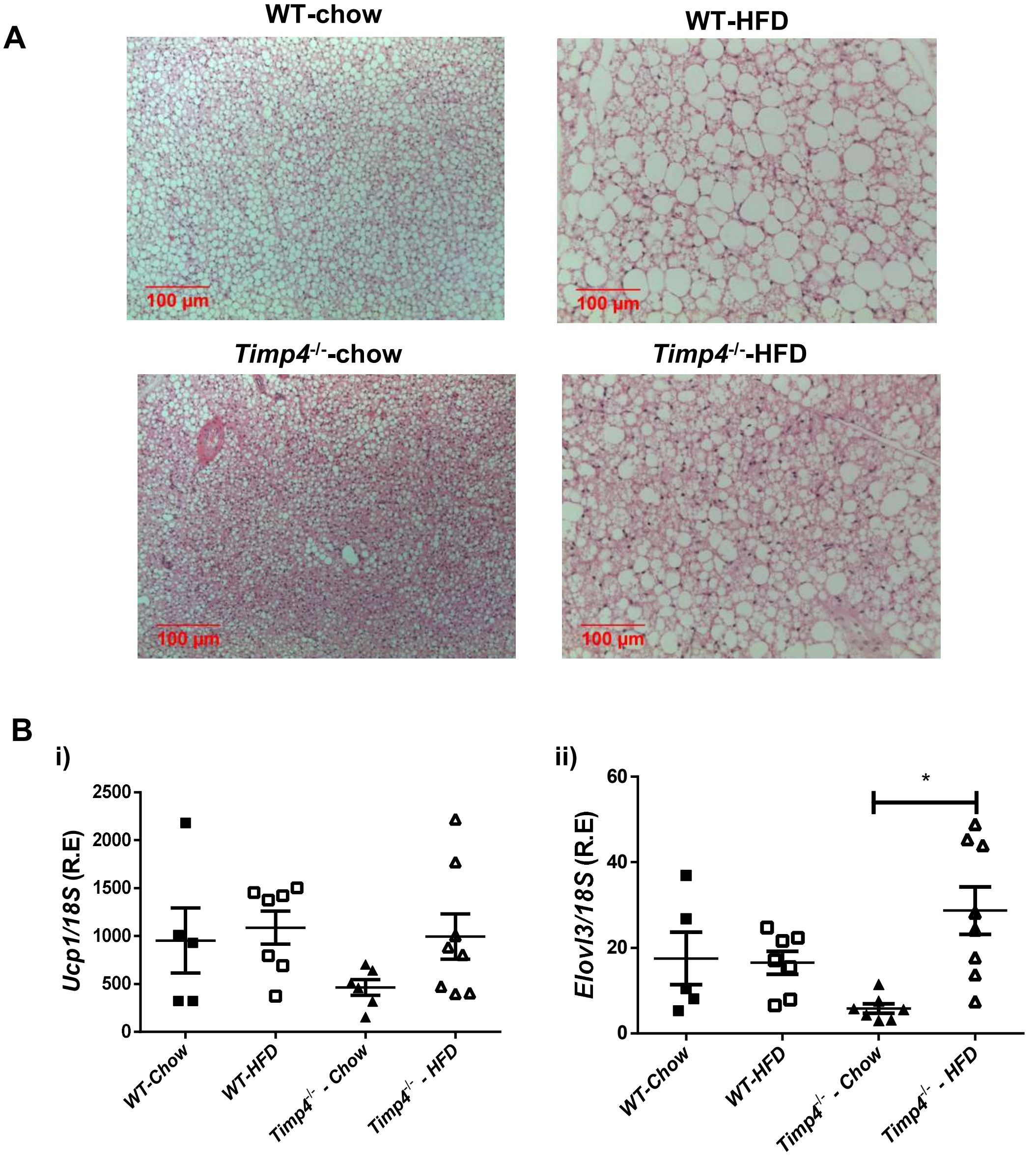

Supplementary Figure 5. ***Timp4*** **gene deletion does not alter** ***Ucp1*** **levels in brown adipose tissue.** (A) H&E stained brown adipose tissue in chow- and HFD-fed mice of each genotype. (B) mRNA expression of *Ucp1* (i) and *Elovl3* (ii) in indicated groups. 18S rRNA was used as internal control. Data were represented as Mean ± S.E and analyzed by ANOVA. * indicates significance ($p<0.05$).

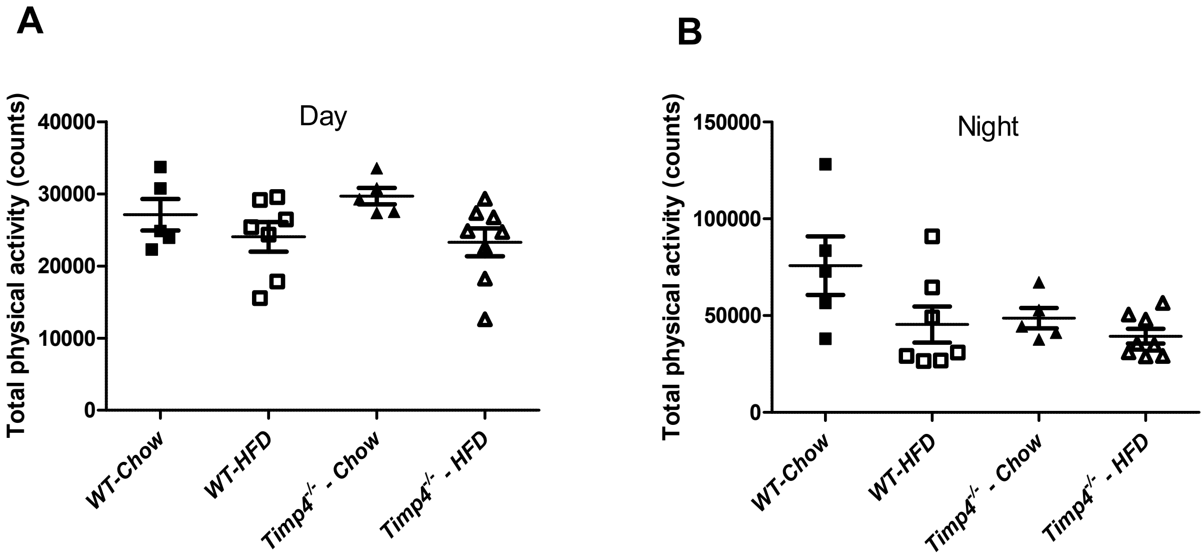

Supplementary Figure 6: *Timp4* gene deletion does not alter physical activity. Physical activity of chow-fed and HFD-fed WT and *Timp4*$^{-/-}$ mice were measured in metabolic cages according to the number of movements along X-, Y- and Z-axis. (A) Physical activity during day. (B).Physical activity during night. Data are presented as mean ± S.E.M and analyzed by ANOVA. * indicates significance ($p<0.05$).

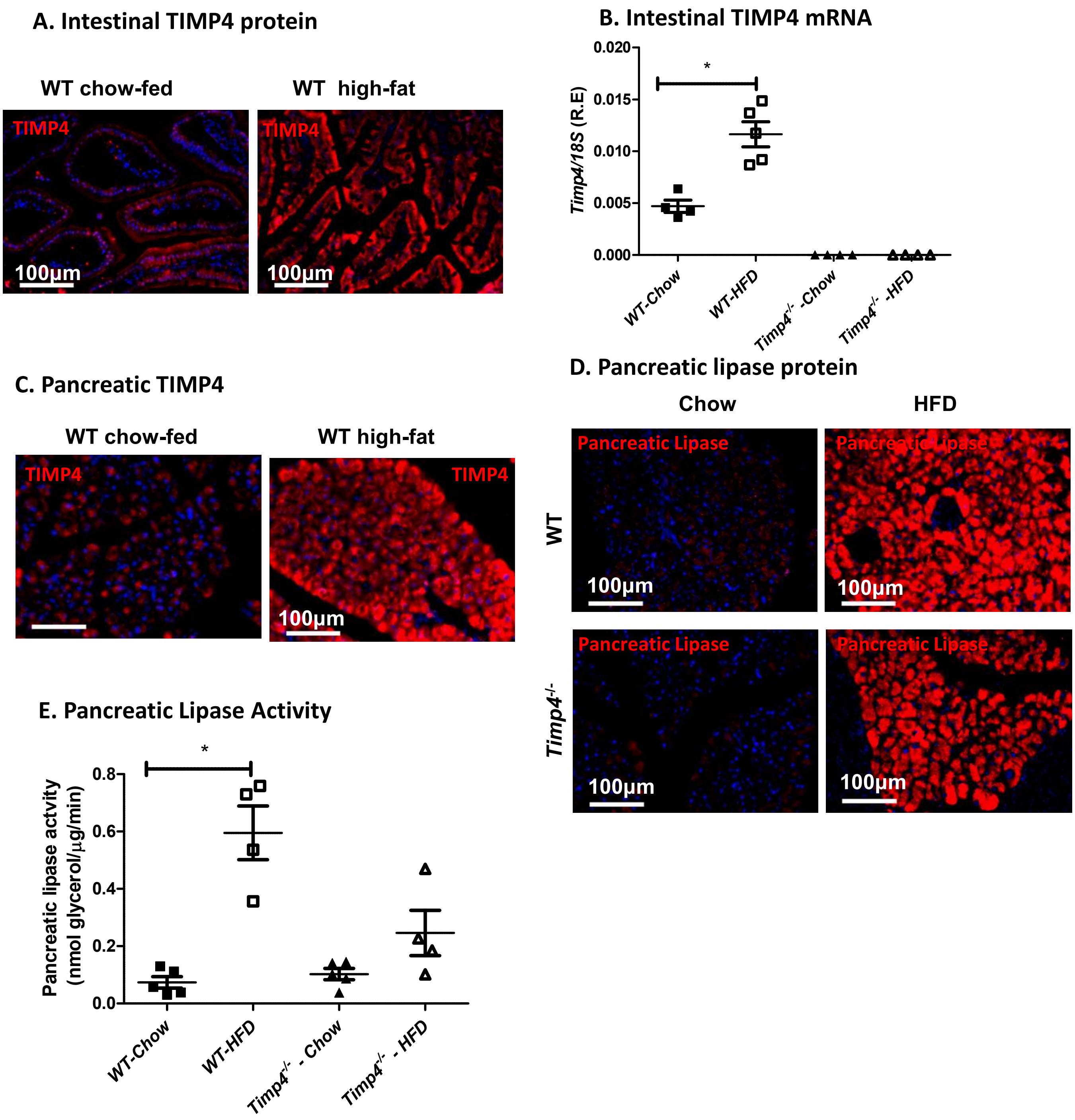

Supplementary Figure 7R1: HFD-induced TIMP4 expression in small intestine and pancreas. Immunofluorescent staining for TIMP4 (A), and *Timp4* mRNA levels in the intestine (enterocytes) (B). Immunofluorescent staining for pancreatic TIMP4 levels (C), and pancreatic lipase protein levels (D).
E) Pancreatic lipase activity in indicated groups. Data are represented as mean ± S.E.M and analyzed by ANOVA. * indicates statistical significance ($p<0.05$).
